# A Study of the Shock Sensitivity of Energetic Single Crystals by Large-Scale Ab Initio Molecular Dynamics Simulations

**DOI:** 10.3390/nano9091251

**Published:** 2019-09-03

**Authors:** Lei Zhang, Yi Yu, Meizhen Xiang

**Affiliations:** 1 CAEP Software Centre for High Performance Numerical Simulation, Beijing 100088, China; 2 Laboratory of Computational Physics, Institute of Applied Physics and Computational Mathematics, Beijing 100088, China

**Keywords:** BTF, TATB, CL-20, cocrystal, energetic materials, shock sensitivity, large-scale ab initio molecular dynamics simulations

## Abstract

Understanding the reaction initiation of energetic single crystals under external stimuli is a long-term challenge in the field of high energy density materials. Herewith, we developed an *ab* *initio* molecular dynamics method based on the multiscale shock technique (MSST) and reported the reaction initiation mechanism by performing large-scale simulations for the sensitive explosive benzotrifuroxan (BTF), insensitive explosive triaminotrinitrobenzene (TATB), four polymorphs of hexanitrohexaazaisowurtzitane (CL-20) pristine crystals and five novel CL-20 cocrystals. A theoretical indicator, *t_initiation_*, the delay of decomposition reaction under shock, was proposed to characterize the shock sensitivity of energetic single crystal, which was proved to be reliable and satisfactorily consistent with experiments. We found that it was the coupling of heat and pressure that drove the shock reaction, wherein the vibrational spectra, the specific heat capacity, as well as the strength of the trigger bonds were the determinants of the shock sensitivity. The intermolecular hydrogen bonds were found to effectively buffer the system from heating, thereby delaying the decomposition reaction and reducing the shock sensitivity of the energetic single crystal. Theoretical rules for synthesizing novel energetic materials with low shock sensitivity were given. Our work is expected to provide a useful reference for the understanding, certifying and adjusting of the shock sensitivity of novel energetic materials.

## 1. Introduction

Energetic materials (EMs) such as explosives, oxidizers and propellants are of significant importance in aerospace, oil-well drilling and other military and civilian applications. In this field, understanding the sensitivity of single crystals under shock or impact has long been a challenge. In engineering, shock sensitivity can be identified by the shock initiation threshold pressure, *P*_90_, which is obtained from the gap test and produces a detonation 50% of the time. *P*_90_ results are generally reproducible and reliable. However, because of the high complexity of the test, the measurements have been performed for only limited types of energetic single crystals [1,2] and the test is not easily applicable to newly synthesized EMs. On the other hand, the drop-weight impact test, which characterizes the impact sensitivity of EMs by height *h*_50%_, is easy to implement. Therefore, there are abundant values of *h*_50%_ in the literature compared to *P*_90_ values. However, the *h*_50%_ value is generally not reproducible as the results significantly vary depending on the conditions under which the tests are performed. For example, for benzotrifuroxan (BTF), the reported *h*_50%_ values vary from 21 [3] to 50 [4] cm; for hexanitrohexaazaisowurtzitane/trinitrotoluene (CL-20/TNT) cocrystal, the values vary from 30 [4] to 99 [5] cm. Therefore, the *h*_50%_ values derived from the same experiments are comparable, while those from different equipment can only be used for a quantitative comparison of the mechanical sensitivity among various energetic single crystals.

With the rapid increase of computational capability and the development of material modeling methods, large-scale atomistic simulation becomes a powerful tool for understanding the physical processes of materials under extreme conditions [6,7]. However, in recent decades, there has been a strong tendency in the literature to elucidate the sensitivity of energetic single crystals by the electron density properties in a separate molecule of EM, such as electrostatic potential, molecular electronegativities, partial atomic charges, molecular weights, vibrational states, oxygen balance of the molecules, detonation gas concentrations and heats of detonation [1]. These quantities ignore the deformation of the chemical bonds, the motion of the molecules, the inner/inter molecular chemical reactions and the symmetrical structure of the crystals, and thereby cannot comprehensively characterize the reaction initiation of an energetic single crystal under shock.

To this end, we developed an *ab initio* molecular dynamics method [8] and extended its computational capability by improving the code’s parallel calculation efficiency. We performed shock wave simulation tests on 11 types of energetic single crystals, with each simulated model composed of ~1000 atoms. On the basis of the calculations, we proposed a theoretical indicator to characterize the shock wave sensitivity of energetic single crystals, which is expected to be useful for the evaluation and adjustment of the shock sensitivity of novel EMs. We also revealed the shock reaction initiation mechanism, found factors that can inhibit the shock sensitivity of EMs and provided theoretical rules for synthesizing novel EMs with low shock sensitivity.

## 2. Methodology

### 2.1. Multiscale Simulation Method of Shock Wave Tests

**Figure 1:**
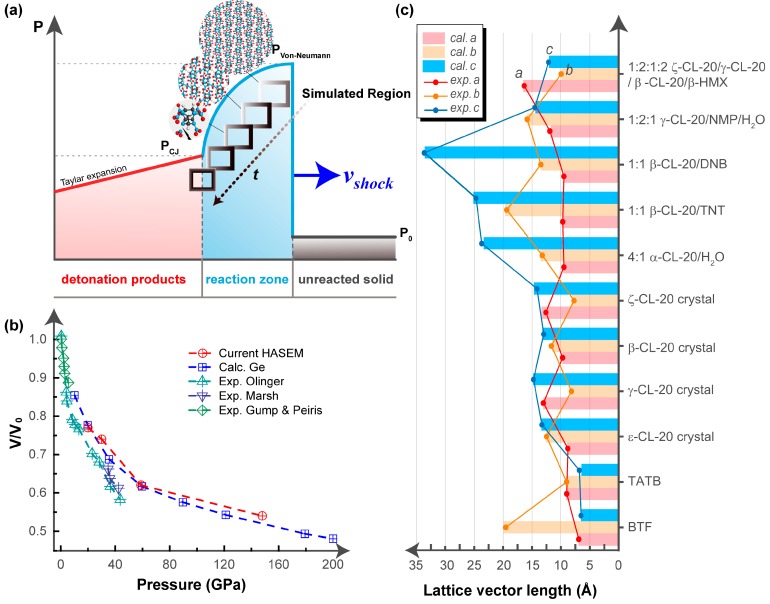
Dynamics simulation method of shock wave tests. (**a**) Schematics of the simulation method. (**b**) Validation of the code by comparing the Hugoniot curve of a single crystal model of octogen (HMX) single crystal to the reported experimental and calculational results in the literature. (**c**) Accuracy verification of the code by comparing the lattice lengths of the studied eleven energetic single crystals to the experimental values obtained by X-ray crystallography.

Figure 1a schematically shows the simulated dynamical shock process in a single crystal. At the beginning when the shock wave reaches the single crystal, the simulation region starts to undergo lattice and molecular deformation. With the increase of simulation time, the simulation region gradually leaves the wave front relative to the unshocked material. The molecules in the simulated region are then compressed to react, eventually reaching the Chapman–Jouguet (CJ) state when the shock wave propagates steadily. We implemented the multiscale shock technique (MSST) [9,10] in the High Accuracy atomistic Simulation for Energetic Materials (HASEM) package [8], so as to capture the atomic motions and the chemical reactions of inner/inter molecules on the density functional theory (DFT) accuracy level.

#### 2.1.1. Continuum Theory Description of the Shock Wave Structure

The shock wave propagation was modelled using the one-dimensional Euler equations for compressible flow [9,10], which were represented by the conservation of mass, momentum, and energy, respectively, in the regions before and after the shock wave interface.

(1) $$u=v_{shock}(1-\frac{\rho_{0}}{\rho }),$$

(2) $$p-p_{0}=v_{shock}{}^{2}\rho_{0}(1-\frac{\rho_{0}}{\rho }),$$

(3) $$e-e_{0}=p_{0}(\frac{1}{\rho_{0}}-\frac{1}{\rho })+\frac{v_{shock}{}^{2}}{2}{\left(1-\frac{\rho_{0}}{\rho }\right)}^{2},$$

where $v_{shock}$ is the shock wave speed, *u* is the particle velocity, *ρ* is the material density, *e* is the energy per unit mass and *p* is the negative component of the stress tensor along the shock propagation direction. The quantities with 0 subscript describe the states of the unreacted material.

#### 2.1.2. Molecular Dynamics Description of the Atomic Motions

In the simulated region, the atomic motions were simulated using the molecular dynamics (MD) method. The Lagrangian per unit mass is

(4) $$L=T_{e}(\{{\dot{\vec{r}}}_{i}\})-V_{e}(\{{\vec{r}}_{i}\})+\frac{1}{2}Q{\dot{\upsilon }}^{2}+\frac{1}{2}\frac{v_{shock}{}^{2}}{\upsilon_{0}{}^{2}}{\left(\upsilon_{0}-\upsilon \right)}^{2}+p_{0}(\upsilon_{0}-\upsilon ),$$

where υ=1/ρ is the specific volume; *T_e_* and *V_e_* are kinetic and potential energies, respectively, and their sum equals to *e*; *Q* is a parameter related to the mass of the simulated cell. When the volume of the simulated cell was fixed, that is, when $\dot{\upsilon }=0$, the Lagrangian expression is equivalent to the continuum Hugoniot relation of Equation (3).

The atomic position and velocity at each time step were obtained from the control equation of the simulated cell volume:

(5) $$Q\ddot{\upsilon }=\frac{\partial T}{\partial \upsilon }-\frac{\partial V}{\partial \upsilon }-p_{0}-\frac{v_{shock}{}^{2}}{\upsilon_{0}{}^{2}}(\upsilon_{0}-\upsilon ),$$

which degenerates to the Rayleigh line of Equation (2) when the cell volume changes uniformly [9,10]. Thus, the system is restrained to fit the shock Hugoniot and the Rayleigh line of the material by changing the volume of the simulated cell.

#### 2.1.3. Density Functional Theory Description of the Electronic Structure

The atomic force of each time step was updated according to the DFT calculations of the electronic structure using HASEM software. The generalized gradient approximation was used for the exchange-correlation functional in the Perdew–Burke–Ernzerhof form. Norm-conserving pseudopotentials specialized for EM crystals were used to replace the core electrons. The valence electrons, described by linear combinations of numerical pseudoatomic orbitals, were calculated on a three-dimensional real-space grid. The reliability of the DFT calculations to describe the structures, energetics, dynamics, mechanical properties, detonation performance and sensitivity of EM crystals has been extensively confirmed in previous work [8,11,12,13,14,15].

In order to improve the computational capability of the dynamics simulation method, we reconstructed the HASEM software based on the J parallel adaptive structured mesh applications infrastructure (JASMIN), which has successfully accelerated many parallel programs for large scale simulations of complex applications on parallel computers [16]. Through this, the calculation efficiency of HASEM software was improved by one order of magnitude. Simulations of large-scale systems containing ~1000 atoms can thereby be achieved by using extended central processing units (CPUs) on supercomputers.

#### 2.1.4. Verification and Validation of the Dynamics Simulation Method

We constructed a single crystal model of octogen (HMX) and performed shock wave tests using the newly developed dynamics simulation method. A series of shock waves, with a speed smaller than 5 km/s, were applied on the HMX model. As shown in Figure 1b, the obtained Hugoniot curve satisfactorily agreed with the experiments [17,18,19] and other calculations [20], thereby confirming the reliability of the current method.

### 2.2. Simulation Models of Eleven EM Single Crystals

CL-20 has been proven to show excellent performance since it was first synthesized by the Naval Air Warfare Center China Lake 30 years ago [21]; however, it has not been widely used until now because of the sensitivity problems of its ε, β, γ and ζ polymorphs [21]. Cocrystallization, which mixes several components on a molecular level, has been considered a promising technique to obtain advanced EMs with good detonation performance and low sensitivity to accidental initiation. Under that circumstance, five novel cocrystals—CL-20/H_2_O, CL-20/TNT, CL-20/1,3-dinitrobenzene (CL-20/DNB), CL-20/N-methyl-2-pyrrolidone/H_2_O (CL-20/NMP/H_2_O) and CL-20/HMX, have been recently synthesized.

Herewith, we studied the four CL-20 polymorphs and the five novel CL-20 cocrystals, as well as the sensitive explosive BTF and the insensitive explosive triaminotrinitrobenzene (TATB). For the 11 EMs studied, we optimized the crystal geometries using the conjugate gradient method on a DFT level, with the initial inputs taken from the lattice parameters and atomic coordinates from single-crystal X-ray diffraction analysis [5,22,23,24,25,26,27,28,29]. The structures were considered as optimized when the stress components were less than 0.01 GPa and the residual forces were less than 0.03 eV/Å. As shown in Figure 1c, the calculated lattice lengths showed satisfactory agreement [30] with the experimental measurements (σ = 0.18 Å; *R*^2^ = 0.9992), thereby confirming the reliability of the current method.

Subsequently, we built large-scale supercells of the eleven EM crystals for the shock simulations. As shown in Table 1, the supercells generally contained more than 1000 atoms, with the lattice length in the range of 15~30 Å. There were 24~64 molecules in each supercell, containing 72~192 trigger chemical bonds. The number of the chemical bonds included here was an order of magnitude larger than the traditional *ab initio* MD simulations, and thereby better reflects the randomness and probability characteristics of the chemical reaction kinetics.

### 2.3. Control Parameter of the Shock Simulation Tests

All the 11 systems were shocked with the same speed *V_shock_* = 9 km/s at a direction perpendicular to the lattice vector. We used this shock wave speed based on a previous classical MD study, in which the breaking of the CL-20 trigger bonds was apparent at this shock speed [31]. The time step for the *ab initio* MD simulation was set to be 0.1 fs. As shown in Table 1, under this shock condition, 10 of the systems (excluding the insensitive explosive TATB) were initiated within 10,000 steps, that is, 1000 fs.

We defined the chemical bonds as broken when they were stretched to a cutoff percentage relative to each equilibrium state. The cutoff criterion was 20% on average, but it varied from 10% to 40% for different types of bonds. The criterion for each type of bond was determined by the statistics of the reaction products of the TATB explosive when the number of product molecules best agreed with the number of the stable clusters with a life span more than hundreds of time steps during the kinetics simulation.

## 3. Results and Discussion

### 3.1. Shock Dynamics of the 11 EM Crystals

**Figure 2:**
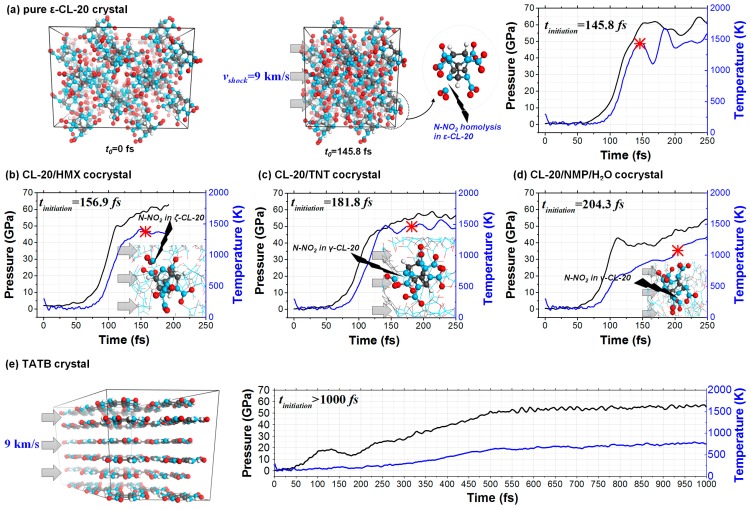
Shock dynamics of the (**a**) ε-CL-20 crystal, (**b**) CL-20/HMX cocrystal, (**c**) CL-20/TNT cocrystal, (**d**) CL-20/NMP/H_2_O cocrystal and (**e**) TATB crystal. In each panel, the temperature and pressure are shown as a function of time. The molecular conformations at the beginning of each decomposition reaction are also plotted.

The 11 crystals studied were rapidly compressed under shock, as shown in Figure 2. The temperature and pressure of the systems increased as a function of time during the shock process. The molecules were packed more densely in space and the chemical bonds plastically deformed to break, leading to the decomposition of material. According to our simulation, the N–NO_2_ bond, N–O bond and C–NO_2_ bond were the most active chemical bonds to deform and break in the CL-20 molecule, BTF molecule and TATB molecule, respectively, under shock. We therefore denoted these bonds as the “trigger bonds”.

**Figure 3:**
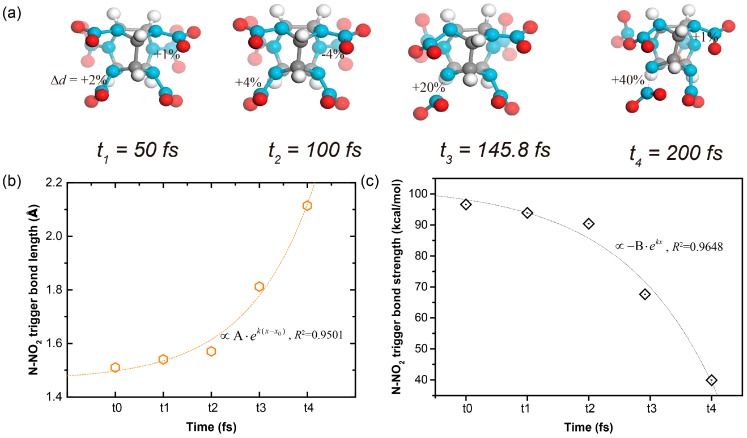
(**a**) Conformation vs time plots for a molecule in the shocked ε-CL-20 crystal, along with the corresponding (**b**) length and (**c**) strength of the trigger bond N–NO_2_ in each snapshot of (**a**).

Take the shocked ε-CL-20 crystal as an example. The N–NO_2_ bonds with the exo-spatial orientation with respect to the five-membered imidazolidine ring were the trigger bonds. During the shock process, the molecular conformations at different times are shown in Figure 3a. Both the increase of the trigger bond length and the decrease of the trigger bond strength went in an exponential manner, as shown in Figure 3b,c. When the stretching of the chemical bond reached the cutoff percentage relative to the equilibrium state, we defined it as broken. Therefore, the first breaking of the trigger bond of the shocked ε-CL-20 crystal occurred at *t*_3_ = 145.8 fs.

**Figure 4:**
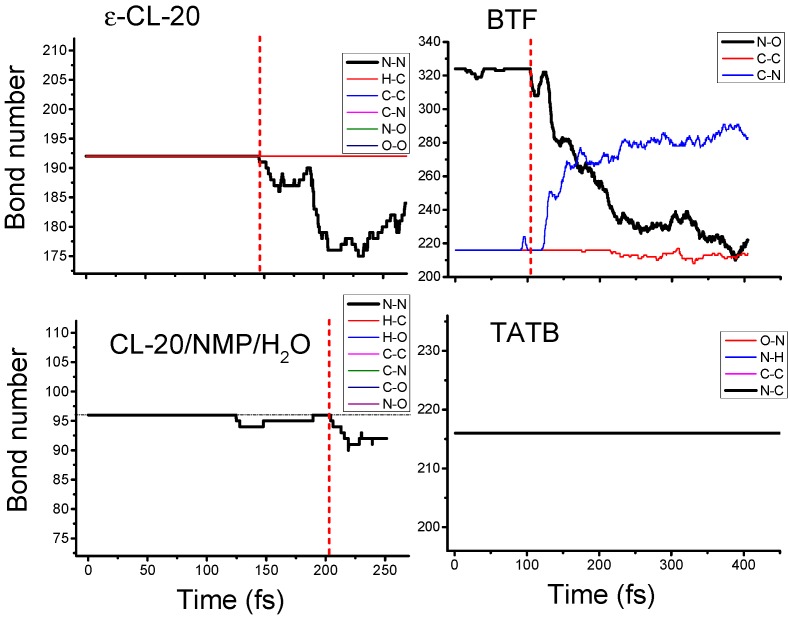
Bond number vs time plots for the shocked ε-CL-20 crystal, BTF crystal, CL-20/NMP/H_2_O cocrystal and TATB crystal. The chemical bonds counted included N–N, H–C, H–N, H–O, C–C, C–N, C–O, N–O and O–O bonds. The sign of the initiation of the reaction under shock was a continuous reduction in the number of trigger bonds.

The chemical bonds of the eleven crystals studied included N–N, H–C, H–N, H–O, C–C, C–N, C–O, N–O and O–O bonds. For all types of chemical bonds in each crystal, we counted their number as a function of time during the shock process. As shown in Figure 4, the covalent bonds’ breaking and recombination of shocked material was a dynamical process. For example, the trigger bonds N–NO_2_ in CL-20/NMP/H_2_O started to break at 124.8 fs, but they recombined to the initial state at 189.3 fs. We thereby defined the initiation of the chemical reaction by the time *t_initiation_*, from when the breaking of the chemical bonds was always more than their recombination and the number of the trigger bonds decreased continuously. Therefore, the decomposition reaction began at *t_initiation_* = 103.6 fs for BTF and at 204.3 fs for CL-20/NMP/H_2_O.

Generally speaking, for the energetic crystals containing CL-20 molecules, both the temperature and the pressure increased slowly at the beginning of the shock process, as shown in Figure 2. Then, at ~100 fs, the temperature and the pressure started to drastically increase to higher than 1000 K and higher than 50 GPa. Next, at ~150 fs, the systems reached a gently varied stage, during which the trigger chemical bonds started to break and the decomposition reactions of material began. For the shocked insensitive explosive TATB, both temperature and pressure varied uniformly, and no chemical reactions occurred before 1000 fs, while for the shocked sensitive explosive BTF, the chemical reaction already started at 103.6 fs.

### 3.2. Theoretical Indicator of Shock Sensitivity: t_initiation_

Because of the lack of experimental shock sensitivity for most of the EMs studied, we proposed using *t_initiation_* as a theoretical indicator to characterize the ease of the shock reaction initiation. Because shock sensitivity has been proven to have a satisfactory correlation with the impact sensitivity [1,2], we used the experimental value *h*_50%_ as a reference to compare with *t_initiation_*, as shown in Table 2. We note comparing *h*_50%_ values from the same experiments can well reflect the relative sensitivity of different compounds, while comparing those from different experiments was only qualitatively reasonable because of the influence of different experimental conditions used.

As shown in Table 2, there was a satisfactory agreement between the *t_initiation_* and the *h*_50%_ values derived from the same experiment. For example, in experiment 1, BTF had the highest sensitivity with *h*_50%_ = 50 cm, and TATB had the lowest sensitivity with *h*_50%_ > 320 cm. Correspondingly, BTF had the shortest delay of shock reaction at *t_initiation_* = 103.6 fs, while TATB had the longest delay with *t_initiation_* > 1000.0 fs. In experiment 2, the sensitivity order characterized by *h*_50%_ was ε-CL-20 > CL-20/H_2_O > CL-20/NMP/H_2_O. Correspondingly, the sensitivity order quantified by *t_initiation_* was exactly the same, which was 145.8 fs for ε-CL-20, 174.4 fs for CL-20/H_2_O and 204.3 fs for CL-20/NMP/H_2_O. In experiments 3 and 4, the *h*_50%_ for ε-CL-20 was less than those for CL-20/HMX and CL-20/TNT. Consistent with this, *t_initiation_* = 145.8 fs for ε-CL-20 was also smaller than *t_initiation_* = 156.9 fs for CL-20/HMX and *t_initiation_* = 181.8 fs for CL-20/HMX.

According to all the above comparisons between the calculated *t_initiation_* and the measured *h*_50%_ values, *t_initiation_* is a reproduceable and reliable indicator to calibrate the shock sensitivity.

### 3.3. Mechanism of Shock Reaction Initiation

**Figure 5:**
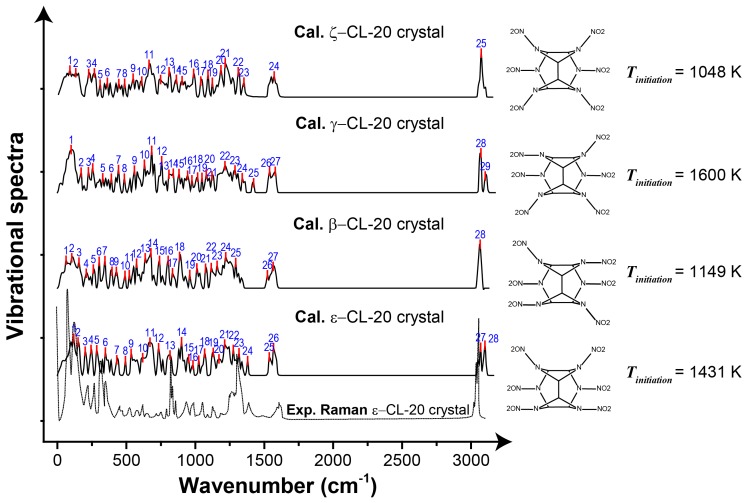
Vibrational spectra for ε, β, γ and ζ polymorphs of CL-20 crystals. The characteristic peaks of the vibrational spectra are marked by vertical red lines and are indexed by blue texts. For each of the four polymorphs, the molecular conformation and the temperature (*T_initiation_*) the crystal starts to decay under shock are also shown.

The shock can be simplified into a perfect impulse *f(t)*, which has an infinitely small duration. Its Fourier transform $F(\omega )=\int_{-\infty }^{+\infty }f(t)e^{-j\omega t}dt=F_{0}$ implies that the shock causes a constant amplitude response in the entire frequency domain. Therefore, the more characteristic peaks in the vibrational spectra of an energetic crystal, the more modes can be excited and the more heat can be generated under the same shock condition. We plotted the vibrational spectra for the ε, β, γ and ζ polymorphs of CL-20 crystals in Figure 5. The number of characteristic peaks of the vibrational spectra of ζ-CL-20 was 25, which was the least among the four polymorphs. Correspondingly, the generated temperature *T_initiation_*= 1048 K was also the lowest. On the other hand, that peak number was the most for γ-CL-20 (29) and consistent with this, the temperature of *T_initiation_* = 1600 K was the highest. For the other two polymorphs, both the peak number and the temperature fall in the middle.

In order to study the mechanism of the initiation of shock reaction, we calculated for the 11 crystals the strength of the trigger bond *S_trigger_* and the temperature rising rate (TRR) under shock, as shown in Table 2. In this, the bond strength was quantified by the integrated value of the crystal orbital Hamilton population (COHP) at band energy, and the temperature rising rate was calculated by dividing the temperature (when t = *t_initiation_*) by *t_initiation_*.

**Figure 6:**
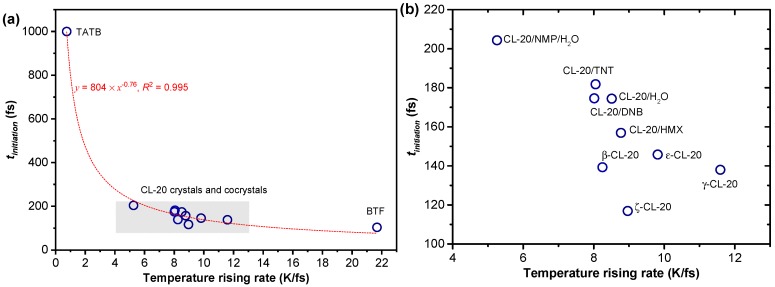
(**a**) Correlation between the shock sensitivity and the temperature rising rate under shock; (**b**) is an enlarged plot in a focused range, as marked by gray in plot (**a**).

As shown in Table 2, the trigger bond strength of the sensitive explosive BTF was *S_trigger_* = 42 kcal/mol, while that of the insensitive explosive TATB was three times higher. As well as this, the temperature rising rate of shocked BTF was *TRR* = 21.7 K/fs, while that of TATB was 29 times smaller. For the other EMs containing CL-20 molecules, both the trigger bond strength and the TRR fell in the range between BTF and TATB. In addition, we found that the *t_initiation_****–****TRR* correlation showed a satisfactory power function $y=804\;\times x^{-0.76}$, with the coefficient of determination *R*^2^ = 0.995, as shown in Figure 6. Therefore, the ease of the shock reaction initiation was apparently determined by the trigger bond strength and the temperature rising rate under shock. As a simplification, the specific heat capacity of a compound was the amount of heat needed per unit mass in order to raise the temperature by ΔT. Therefore, a compound with a larger specific heat capacity generally has a smaller TRR. We thereby propose that stronger covalent bonds and higher specific heat capacity are beneficial for delaying the time of shock initiation ***t_initiation_***, that is, reducing the shock sensitivity.

Among the five CL-20 cocrystals, CL-20/NMP/H_2_O had the lowest TRR = 5.3 K/fs, while the other EMs had their TRR values in the range of 8.0–8.8 K/fs, as shown in Figure 6b. At the same time, the trigger bond strength of CL-20/NMP/H_2_O was also the highest, which was *S_trigger_* = 115 kcal/mol, as shown in Table 2. Therefore, CL-20/NMP/H_2_O was able to obtain the lowest shock sensitivity among the five cocrystals.

From the explanation above, the initiation of the shock reaction of energetic single crystals is shown to be a process driven by the coupling of heat and pressure. The heat is derived from the mechanical work of the shock compression and is transferred into the vibration of the lattice, the molecules and the chemical bonds of the shocked material. Denser characteristic peaks of vibrational spectrum correspond to a larger amount of heat generated by the shock. Driven by the heat, the temperature of the system quickly increases and the stretch vibrational modes of the chemical bonds are activated. While vibrating, the chemical bonds also endure plastic deformation under the shock compression. When the deformation of the trigger bond is beyond the critical level, the shock reaction begins.

### 3.4. Shock Sensitivity Buffer: Intermolecular Hydrogen Bond

On the basis of the calculated ***t_initiation_***, we were able to predict the relative sensitivity of all the 11 EMs studied, which was shown to be BTF > ζ-CL-20 > γ-CL-20 > β-CL-20 > ε-CL-20 > CL-20/HMX > CL-20/H_2_O > CL-20/DNB > CL-20/TNT > CL-20/NMP/H_2_O > TATB. The predicted order shows a close relationship between the shock sensitivity and the hydrogen bonding amount. For example, BTF contains no hydrogen and it owns the highest sensitivity, whereas TATB contains the most hydrogen and it has the lowest sensitivity.

**Figure 7:**
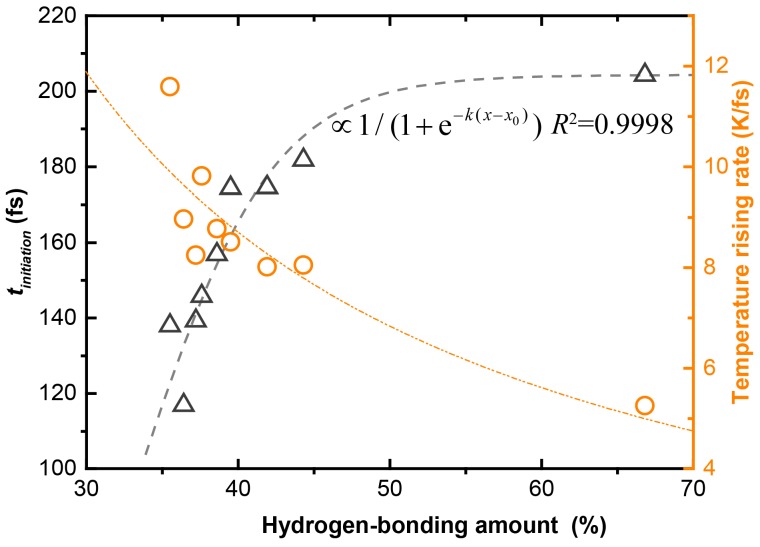
Shock sensitivity vs amount of hydrogen bonding for the CL-20 composed EMs studied.

In Figure 7 we show quantitatively the relationship between the shock sensitivity and the hydrogen bonding amount for the EMs containing CL-20 molecules, wherein the hydrogen bonding amount is represented by the occupied percentage in the Hirshfeld surface of the CL-20 molecules. The correlation is a satisfactory exponential function, in which $t_{initiation}\propto 1/\left(1+e^{-k\left(x-x_{0}\right)}\right)$, with the coefficient of determination *R*^2^ = 0.9998. The correlation implies that the more hydrogen bonding occurs, the lower the shock sensitivity.

The intermolecular hydrogen bond A:H–D (with “:” representing the electron lone pair, A for acceptor and D for donor) integrates the H–D polar-covalent bond, the A:H nonbond, and the A–D repulsive coupling interaction. Under shock, the hydrogen bonds show their elasticity—the covalent bond segment contracts and the nonbond elongates [33,34]. The special elasticity allows hydrogen bonds to vibrate in a continuous frequency region (<200 cm^−1^) so that the crystal can absorb more energy from the shock before reaching a temperature that is too high. This is analogous to the function of hydrogen bonds on improving the specific heat capacity of liquid H_2_O [35]. In order to confirm our hypothesis, we show the relationship between the TRR and the hydrogen bonding amount in Figure 7, which is roughly a power function. This relationship suggests that the hydrogen bonding has a buffering effect on the heating of the system under shock, thereby delaying the initiation time of the chemical reaction *t_initiation_*. This is the fundamental reason why cocrystallization with low-sensitive EM components can effectively reduce the sensitivity of CL-20 crystals.

## 4. Conclusions

To conclude, we have developed an *ab initio* molecular dynamics method based on the multiscale shock technique and performed shock wave simulation tests for the sensitive explosive BTF, insensitive explosive TATB, four polymorphs of CL-20 crystals and five novel CL-20 cocrystals, with each model containing ~1000 atoms. The main conclusion includes:

(1) We proposed a theoretical indicator *t_initiation_* to characterize the shock sensitivity of an energetic single crystal, which has been proven to be reliable and satisfactorily consistent with experiments.
(2) The shock reaction initiation was found to be a process driven by heat and pressure coupling and the vibrational spectra, the specific heat capacity, as well as the strength of the trigger bonds being the determinants of the shock sensitivity of energetic single crystals.
(3) Intermolecular hydrogen bonds were found to effectively buffer the system from heating, thereby delaying the trigger bonds from breaking and ultimately reducing the shock sensitivity of the energetic crystal.
(4) To synthesize advanced energetic materials with low shock sensitivity, small characteristic peak density of the crystal vibrational spectra, high specific heat capacity, strong trigger chemical bonds and high hydrogen bond amounts were theoretically recommended.

Our work is expected to provide a theoretical reference for the understanding, certifying and adjusting of the mechanical sensitivity of the single crystals of novel energetic materials.

## Figures and Tables

**Table 1:** Lattice lengths (Å) of the supercells used for shock simulations. The type of the trigger bonds for each crystal, as well as the number of the composed atoms, molecules and trigger bonds in each supercell, are also given. EMs = energetic materials, BTF = benzotrifuroxan, TATB = insensitive explosive triaminotrinitrobenzene, CL-20 = hexanitrohexaazaisowurtzitane, TNT = trinitrotoluene, HMX = single crystal model of octogen.

EMs	*a*	*b*	*c*	Trigger Type	Number of		
					Atoms	Molecules	Triggers
**Sensitive**							
BTF	20.86	19.89	19.63	N–O	648	36	108
**Insensitive**							
TATB	18.18	27.31	19.44	C–N	576	24	72
**Pure CL-20**							
ε-polymorph	17.83	25.26	26.70	N–N	1152	32	192
γ-polymorph	26.11	16.75	29.66	N–N	1152	32	192
β-polymorph	19.47	23.03	26.44	N–N	1152	32	192
ζ-polymorph	26.74	16.22	29.37	N–N	1152	32	192
**CL-20 cocrystal**							
4:1 γ-CL-20/H_2_O	19.15	27.02	23.36	N–N	1176	40	192
1:1 β-CL-20/TNT	19.33	19.65	25.14	N–N	912	32	96
1:1 β-CL-20/DNB	18.94	13.48	33.57	N–N	832	32	96
1:2:1 γ-CL-20/NMP/H_2_O	23.50	15.82	28.88	N–N	1136	64	96
1:2:1:2 ζ-CL-20/γ-CL-20/β-CL-20/β-HMX	16.56	19.81	24.06	N–N	800	24	96

**Table 2:** The experimental *h*_50%_ (cm) values and the initiation time of the shock reaction *t_initiation_* (fs) of the 11 EMs studied. The strength of the trigger bond ***S_trigger_*** (kcal/mol), the temperature *T_initiation_* (K) and the temperature rising rate *TRR* (K/fs) for each crystal are also given for the study of the mechanism of the shock reaction initiation.

	*h*_50%_							*S_trigger_*	*t_initiation_*	*T_initiation_*	*TRR*
EMs	Expt 1 [4]	Expt 2 [27]	Expt 3 [28]	Expt 4 [5]	Expt 5 [3]	Expt 6 [32]	Expt 7 [26]	Current Calculation			
**Sensitive**											
BTF	50				21			42	103.6	2246	21.7
**Insensitive**											
TATB	>320							125	>1000.0	754	0.8
**Pure** **CL-20**											
ε-polymorph		14	29	47		12–21	13	112	145.8	1431	9.8
γ-polymorph								112	138.0	1600	11.6
β-polymorph						14		111	139.3	1149	8.2
ζ-polymorph								110	116.9	1048	9.0
**CL-20 cocrystal**											
CL-20/H_2_O		16						113	174.4	1484	8.5
CL-20/TNT				99			30	112	181.8	1464	8.1
CL-20/DNB							55	111	174.6	1400	8.0
CL-20/NMP/H_2_O		112						115	204.3	1074	5.3
CL-20/HMX			55					112	156.9	1377	8.8
